# Transcription factors Lef1 and Rest stimulate recovery from depressive states

**DOI:** 10.1038/s41386-025-02259-0

**Published:** 2025-10-07

**Authors:** Hajime Yamamoto, Satomi Araki, Ryoma Onodera, Yasuhiro Go, Kentaro Abe

**Affiliations:** 1 Lab of Brain Development, Graduate School of Life Sciences, Tohoku University, Sendai, Miyagi Japan; 2 Graduate School of Information Science, University of Hyogo, Kobe, Hyogo, Japan; 3 Department of System Neuroscience, Division of Behavioral Development, National Institute for Physiological Sciences (NIPS), National Institutes of Natural Sciences, Okazaki, Aichi Japan; 4 Cognitive Genomics Research Group, Exploratory Research Center on Life and Living Systems (ExCELLS), National Institutes of Natural Sciences, Okazaki, Aichi Japan; 5 Division for the Establishment of Frontier Sciences of the Organization for Advanced Studies, Tohoku University, Sendai, Miyagi Japan

**Keywords:** Depression, Pharmacology

## Abstract

The depressive state, a hallmark of major depressive disorder (MDD) and frequently observed alongside various neurological diseases, is often characterized by the dysregulation of multiple genes, reflecting a complex and multifaceted pathology underlying its symptoms. To gain deeper insights into the role of transcription factors (TFs) in mediating depressive states, we performed a detailed analysis of transcription factor activity profiles (TFAPs) in mouse brains, employing a TF-activity reporter battery that enables evaluation of transcriptional activities of multiple TFs and endogenous gene expression patterns within the same samples. This approach identified two critical TFs, T-cell factor/lymphoid enhancer factor (TCF/LEF) and RE1-silencing transcription factor (REST), whose activities were closely associated with a depressive phenotype and the transcriptomic alterations observed in mice subjected to chronic social defeat stress. We also found significant alterations in genes downstream of both TCF/LEF and REST in the brains of human patients with MDD. Pharmacological assessment in mice revealed that the neuropsychiatric agents, lithium and sertraline, modulate TCF/LEF and REST activities both in vitro and in vivo. Genetic and pharmacological manipulations of TCF/LEF and REST activities in stress-exposed mice influenced the recovery from depressive states, with combined modulation of both TFs enhancing therapeutic outcomes. These findings underscore the critical roles of TCF/LEF and REST in driving the transcriptomic changes observed in the brain during recovery from a depressive state. TFAP analysis deepens our understanding of the molecular underpinnings of chronic disorders and offers the potential for novel therapeutic interventions.

## Introduction

Depression is among the most pervasive mental health disorders, characterized by persistent brain dysfunction, causing symptoms such as depressed mood, anhedonia, and fatigue [1, 2]. Despite substantial advances, our understanding of the molecular mechanisms driving depressive states in individuals remains insufficient to establish universally effective remedies for all patients [3–7]. One of the most influential theories concerning the molecular etiology of depression is the monoamine hypothesis [8–10]. However, the therapeutic onset of antidepressants targeting monoamines often manifests only after weeks of treatment [11], implying that their efficacy may depend on sustained brain alterations in addition to the increase in synaptic monoamine concentration. Indeed, studies across various animal models have revealed significant transcriptomic changes in the brains of depressed subjects [12–14], a finding further corroborated by postmortem brain analyses of human patients [15, 16]. These findings suggest that dysregulation of gene expression and its regulatory mechanisms in the brain are implicated in both the pathogenesis of depression and the therapeutic response to treatment.

Delineating the molecular mechanisms that govern recovery from depression is pivotal to both advancing our fundamental understanding of the disorder and designing targeted interventions. Although numerous genes have been linked to depressive phenotypes, their individual contributions remain obscure because intricate gene-gene interactions and gene-environment contingencies confound simple causal inferences. Consequently, disease understanding or therapeutic strategies focused on single genes have yielded only limited success [13, 17–20]. Transcription factors (TFs) represent a more compelling class of regulatory hubs. By binding to diverse genomic regulatory elements, a single TF can coordinate broad, synchronous gene expression programs, and dysregulation of TF-activity is therefore widely implicated in the pathophysiology of complex diseases, including psychiatric disorders [21–26]. From a therapeutic standpoint, TFs are far more tractable targets than each of the vast downstream genes, since modulating TFs can, in principle, reprogram an entire disease-relevant regulatory network. Thus, systematically mapping TF-activity in the depressed brain could illuminate pathogenic mechanisms and guide therapeutic design. The challenge, however, is that TF-activity cannot be inferred reliably from transcript abundance alone. Each gene is coregulated by multiple TFs whose combinatorial, synergistic, and nonlinear interactions create a regulatory logic that cannot be reliably deciphered from downstream transcript levels [27–29]. A methodology that directly quantifies TF-activity in vivo would circumvent these computational ambiguities and provide a direct view of the molecular perturbations underlying depressive pathology, thereby revealing actionable therapeutic targets [30].

Recently, we established TF-activity profiling, which enables the quantitative in vivo assessment of transcriptional activities across multiple TFs using a viral vector-based TF-activity reporter battery [31, 32]. This assay is compatible with transcriptome sequencing, enabling simultaneous measurement of TF-activity and downstream gene expression in the same biological specimen. This integrated multi-omics strategy produces a uniquely comprehensive dataset that directly links the dynamic state of each TF to its corresponding gene expression landscape. By comparing TF-activity and transcriptome signatures across individuals with distinct behavioral or pathophysiological states, this method facilitates a systematic understanding of the molecular basis underlying depression-like states. In this study, we applied this method to investigate changes in TF-activity in the brains of a mouse model for depression, mainly focusing on the prefrontal cortex (PFC), an area frequently affected in depression and implicated in both the manifestation of depression symptoms and the recovery process [18, 33–35].

## Material and Methods

### Animals and animal care

Mice (*Mus musculus*, Slc: ICR) obtained through SLC Japan and bred in our facility were used. Mice in this study were maintained in clear plastic cages in a temperature- and humidity-controlled room with a 12-h light/dark cycle (light on at 8:00 a.m. and off at 8:00 p.m.) and could access standard food and water *ad libitum*. The care and experimental manipulation of animals used in this study were reviewed and approved by the Institutional Animal Care and Use Committee of Tohoku University (2018LsA-012, 2018LsA-013, 2020LsA-003, 2020LsA-004, and 2024LsA-002). All experiments and maintenance were performed following relevant guidelines and regulations.

### Viral vectors

All the viral vectors used in this study were generated in-house, as previously described [31, 32]. Lentiviral vectors (LV) were used for TF activity reporter expression, adeno-associated viral vectors (AAV) of serotypes 2/9 were used for knocking down Lef1 and Rest, and for the TF activity reporter for single-cell analysis. For CRISPR-mediated knockdown of Lef1, we used the gRNA sequence of 5′-CAGCGACCCGTACATGTCAA-3′ and 5′-ACGGAGGCCTGTACAACAAG-3′ targeted against the open reading frame (ORF) of the Mus musculus Lef1 gene. The double-strand DNAs containing the gRNA sequence were synthesized and subcloned into the MluI/SpeI site of pAAV-FullH1TO-SaCa9sgRNAi(CREB)-CMV-TetR-2A-EGFP-KASH-WPRE-shortPA (Addgene, #113702; a gift from Jonathan Ploski) [36], which was designed to express gRNA under the H1 promoter depending on doxycycline (Dox). The recombinant AAV to express SaCas9 was created using plasmid pAAV-EFS-SaCas9-P2A-HAFLAGHA-KASH-pA (Addgene, #113688; a gift from Jonathan Ploski) [36]. To knock down REST expression, we used shRNA targeting the protein-coding region of the mouse Rest gene. The construct for Rest knockdown was derived from the AAV8-hSyn-flex-miR30-eGFP-shCrh (Addgene, #132715; a gif from Robert Messing) [37]. We subcloned the AgeI/AscI fragment of this construct, which corresponds to the shRNA expression cassette, into the pAAV-hSynI-mCherry expression vector that expresses the mCherry gene under a human Synapsin-I promoter. The AgeI/AscI fragment was then replaced by a synthesized DNA sequence, with the shRNA target sequence changed to 5’-GAAATATACAGCGCCAATAAA-3’, resulting in the pAAV-hSyn-CRY-miR30-shRest-WPREpA.

### Drug treatments

For in vitro drug treatment, cultured neurons were treated with drugs starting at 14 div. The cells were treated with sertraline hydrochloride (TCI) or lithium chloride (Nakarai-tesque) for 14–22 h, followed by stimulation with bicuculline methiodide (30 μM, WAKO) and 4-aminopyridine (100 μM, SIGMA) for 2 h. Sertraline, doxycycline hyclate, and lithium chloride were applied to the medium at a final concentration of 2.5 μM, 1 μg/mL, and 50 mM, respectively. Sertraline was dissolved in dimethyl sulfoxide (DMSO, TCI), with the final concentration of DMSO being <0.01%. For in vivo drug administration, drugs were diluted in drinking water and given ad libitum to avoid introducing unnecessary stress through forced administration. The sertraline dissolved in DMSO was diluted to 150 mg/L with water. Either doxycycline hyclate (TCI) or doxycycline hydrochloride n-hydrate (Fujifilm-Wako) and lithium carbonate (Nakarai-tesque) were diluted to 1 mg/mL and 600 mg/L, respectively. Water consumption was monitored, and the drug concentration was adjusted to ensure that the administered dose was equivalent across animals.

### Surgical procedure

Mice expressing TF-activity-reporter constructs in their brains were generated as described before [32, 38]. Briefly, lentiviral vectors harboring the TF activity reporters against six TFs were injected into the ventricle of E15 embryos in utero. Mice were raised to 8 weeks when they were subjected to behavioral experiments. For injecting viral vectors into adult mice, we anesthetized mice over 8 weeks of age with the medetomidine-midazolam-butorphanol mixture (medetomidine 30 μg/mL, All Japan Pharma; midazolam 30 μg/mL, Astellas Pharma; butorphanol tartrate 500 μg/mL, Meiji Seika Pharma; NaCl 118 mM; 500 μL per mouse) [38, 39]. For knocking down the endogenous expression of Lef1 in PFC, AAV2/9-TRE-gRNA(Lef1)-CMV-rtTA-T2A-EGFP produced from the plasmid pAAV-FullH1TO-SaCa9sgRNAi(CREB)-CMV-TetR-2A-EGFP-KASH-WPRE-shortPA, and AAV2/9-EF1a-SaCas9-P2A-HA produced from the plasmid pAAV-EFS-SaCas9-P2A-HAFLAGHA-KASH-pA (Addgene #113688), total ~0.5 μL for each locus, were bilaterally injected at the coordinate from the bregma: anterior, +1.85 mm; lateral, ±0.35 mm; ventral, –1.7 mm. The same coordinates were used for virus injection for the single-cell TF-activity analysis.

### Induction of depression-like state

For applying social defeat stress, male mice over 8 weeks were subjected to 10 consecutive days of social defeat. Since our study used ICR mice for both the stressor (aggressor) and the defeated mice, individuals with high aggression were selected prior to the experiment and used as the stressor. For screening aggressive individuals, adult male mice aged over 8 weeks were socially isolated for more than 4 weeks, and their aggressiveness was measured regarding the frequency and severity of aggressive attacks when they were confronted with an unfamiliar male mouse. We used a blue animal marker (ASONE) on the backs of aggressor mice to aid visual distinction of aggressive individuals. Those aggressors were used to provide social defeat stress. Before the beginning of the social defeat, the subject mice were individually housed for a week to eliminate the influence of the social hierarchy established during group housing. To apply social defeat stress, we used a cage measuring 23 × 34 × 14 cm (CLEA Japan), divided into two compartments with transparent plexiglass and six 1 cm diameter holes. The subject mouse was transferred into the aggressor’s compartment for 5 min, where it experienced physical attacks. Subsequently, the subject was moved into the opposite compartment and cohabited with the aggressor for the following 24 h to induce psychological stress. The next day, the procedure was repeated with a different aggressor in the aggressor’s cage. These processes were repeated for 10 days with a different aggressor each day. The control mice were housed in the same type of separate cage and cohabited with non-stressed control mice for 10 days, with daily transfers and cohabitation with another non-stressed mouse. In some experiments, mice were classified as “susceptible” (SI score <1) and “resilient” (SI score ≥1) according to criteria established in previous studies [40]. In other experiments, mice were not separated into subgroups; instead, all mice subjected to social defeat stress were analyzed together as the “defeat” group.

### Behavioral analysis

Mice raised to 8 weeks were subjected to behavioral experiments. All behavioral analyses were performed in a soundproof booth equipped with a USB camera on its ceiling [41]. Throughout the experiment, their behaviors were recorded using a camera, and their movements were tracked and analyzed with the ANY-maze software (Soldering Inc.). To acclimate them to the environment, mice with their home cages were moved to the experiment booth 30 minutes before the start of tests. We used a 40 × 40 × 40 cm grey plexiglass chamber for social interaction and open field tests. In the social interaction test, a display cage (a circular column of 6 cm diameter, made from transparent plexiglass rods) was placed adjacent to the midpoint of a side of the chamber, and an interaction zone with a radius of 11 cm was set around it. Experimental mice were placed on the opposite side of the interaction zone and allowed to explore freely for 150 seconds. After the mouse was brought back to their home cage, an unfamiliar mouse was put in the display cage. Subsequently, the experimental mouse was allowed to explore again for another 150 seconds. The social interaction rate (SI-rate) was defined by calculating the ratio of the time spent in the interaction zone when the display cage was empty (Mouse (−)) to that when a mouse was placed in the display cage (Mouse (+)). Outliers were excluded from the analysis if the data of SI-rate was larger than 4. For time-course analysis of SI-rate change, the SI-rates were normalized for the average value of the control mice at each day point. In the open field test, we recorded the trajectory of the mouse during 10 minutes of free exploration in that chamber. We measured the time spent in the center zone, defined as a region excluding 6 cm from the outer frame. The brightness at the center zone was 50 lux, and the rest was 30 lux. We evaluated the anxiety-like behaviors of mice based on the total time spent in the center zone. In the sucrose preference test, anhedonia was evaluated based on the amount of consumption of water and 1% sucrose water. Bottles of water or 1% sucrose were attached to the cages of the subjects, and the amounts consumed after 24 h were recorded. The position of the bottles was then reversed, and the amounts consumed after another 24 h were measured. The ratio of the sucrose water consumption to the total liquid intake was subsequently calculated. In some mice, the social interaction test was conducted the day after or before social defeat, and the same individuals were used to analyze changes in sociality over time. The open field test was conducted two days after repeated social defeat or social isolation, and the sucrose water preference test was conducted 48 h before and after that.

### Measurement of TF activity

TF-activity was calculated from the ratio of the expressed mRNA amount of the reporter gene and the corresponding reference gene derived from a single TF-activity-reporter construct. The reference genes are constantly expressed by a phosphoglycerate kinase (PGK) promoter, whereas the expression of reporter genes is induced by the minimal promoter with TF binding sites (TFBSs) against which the transcription factor of interest interacts. By changing the combination of a TFBS and the corresponding reference and reporter gene sets, we can measure the activities of various transcription factors from individual samples [32]. After being subjected to experiments, the mouse brain was placed in ice-cold PBS, and then the anterior part of the cortex, including the PFC region, was rapidly dissected. Because the PFC region was identified by visual inspection, these samples are described as “anterior cortex”, as they contain not only PFC but also for surrounding cortical areas. Total RNAs were purified from the specimen by the thiocyanate-phenol-chloroform extraction method and then converted to cDNAs using RevaTra Ace qPCR RT Master Mix with gDNA Remover (TOYOBO, #FSQ-301). The expression of reporter and reference genes was analyzed by quantitative PCR using GoTaq-qPCR Master Mix (Promega, #A6002), with the real-time PCR system (Light Cycler 480, Roche or CFX384, BioRad). The number of such transgenes was quantified by absolute quantification methods against standard plasmids of pre-quantified concentration. In this study, “TF-activity” refers to the change of mRNA levels of the reporter gene relative to the mRNA levels expressed by reference genes from the TF-activity reporters. Certain TFs, including REST/NRSF, act as transcription suppressors depending on the cell context. However, we define increased REST-activity as an elevation in the reporter-to-reference value ratio. To measure the change in TF-activity across different conditions, sibling mice expressing the same reporter virus were randomly assigned to the control and experimental groups. The activities of TFs were normalized by the average activity of that TF in control mice for each experiment. We analyzed a total of 30 TF activity reporters by varying the mixture of viruses to create the reporter mice. In each experiment, up to six viruses were used. In total, TF activity was measured in 80 control, 73 resilient, and 58 susceptible mice. These data were integrated to create a transcription factor activity profile (TFAP).

### Immunostaining

Brains were perfused with 4% paraformaldehyde (Nakarai Tesque) in PBS and dehydrated with 30% sucrose/PBS for 48 h. The fixed brains were embedded into OCT compound (Sakura fintech), frozen at −80 °C, and sectioned to 40 µm thick using a cryostat (CM1850, Leica). The cells in the culture were fixed with 4% paraformaldehyde in PBS for 20 min. The sections and cultures were permeabilized with 0.1% Triton X-100 (Fujifilm-WAKO) in PBS for 15 min, blocked with 10% Normal Goat Serum (Biowest, #S1810-500) for 30 min, and stained by sequential incubation with primary antibodies diluted with PBS at 4 °C overnight and with Alexa Fluor 488- or Alexa Fluor 555-conjugated secondary antibodies (1:450; ThermoFisher) or NeuroTrace 500/525 (1:250; ThermoFisher, #N21480) for an hour, and 4′,6-diamidino-2-phenylindole (DAPI, 1:400; Dojindo, #28718-90-3). The primary antibodies used were as follows: anti-FLAG (1:2000; SIGMA, #F1804), anti-GFP (1:800; MBL, #M048-3MS), anti-HA (1:400; MBL, #561), anti-LEF1 (1:200; SantaCruz, #sc374412), and anti-REST (1:500; ThermoFisher, #IHC-00141). Fluorescent images were obtained using fluorescent microscopies with a 20 × objective lens (Axio Imager.M2, Zeiss), and a 4 × or 10 × objective lens (BZ9000, Keyence). The number of cells and the intensity of staining were quantified by ImageJ (NIH ImageJ).

### Quantifications and statistical analysis

The alpha score of 0.05 was used to reject the null hypothesis. Data visualization and all statistical analyses were performed using R (v4.2 or v4.3). All statistical tests employed in this study were two-sided. For behavioral experiments, we used Welch’s t-test, Tukey HSD-test, two-way ANOVA, two-way repeated-measures ANOVA followed by Tukey post hoc test, binomial test, and Pearson correlation coefficient. For a series of TF-activity analyses, we used Welch’s t-test, hypergeometric distribution analysis, and Pearson correlation coefficient. For a series of transcriptomic analyses, we employed Welch’s t-test, the Benjamini-Hochberg procedure, paired t-test, Wilcoxon rank sum test, hypergeometric distribution analysis, F-test, Pearson’s correlation coefficient, and the Holm method for multiple comparisons. To measure the effect size for the difference in means for animal behavior following drug treatment, we used Hedge’s g statistic on raw SI-rate scores. The number n represents the biological replicates (either individual mice or cell cultures). All boxplots display the median and the interquartile range.

The following procedures are described in the Supplementary Methods: cell culture, RT-PCR analyses, Transcriptome and TF binding site enrichment analysis, single-nucleus RNA-seq analysis.

## Results

### Transcription factor activity profiling of stress-induced depression-like state

**Fig. 1:**
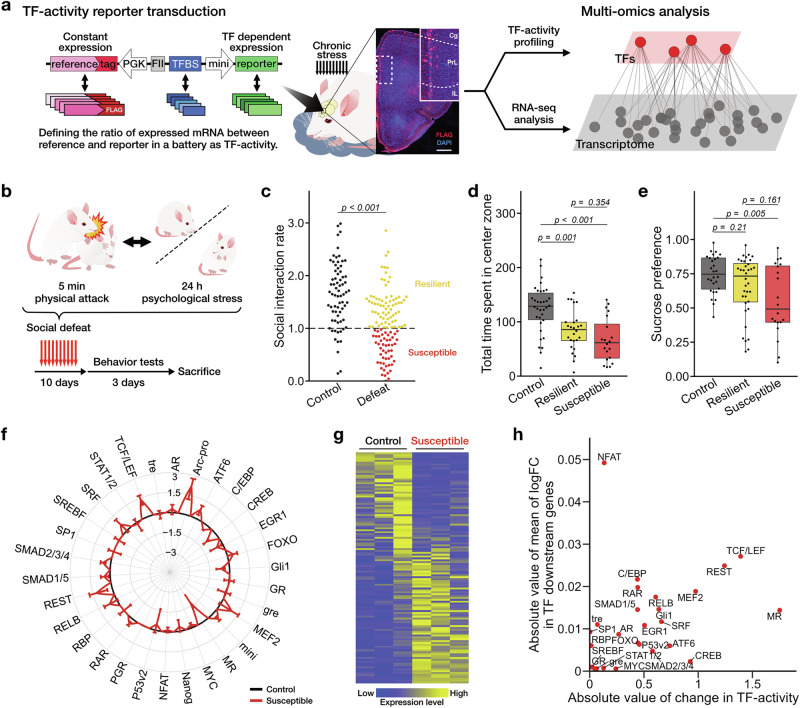
Multi-omics analysis integrating TF-activity profiling and transcriptomic data in stressed mice. **a** Schematic illustration of TF-activity profiling in vivo. The brain image shows the reporter infected cells (red, flag-tag) in the prefrontal cortex. Scale bar, 500 μm. **b** Scheme of repeated social defeat. **c**–**e** Effects of chronic social defeat stress on behaviors. **c** The result of the SI-test for control (black), resilient (yellow), and susceptible (red), Welch’s *t*-test, *n* = 71, 119. **d** The result of the open field test. Tukey’s test; control, *n* = 34; resilient, *n* = 26; susceptible, *n* = 20 mice. **e** The result of the sucrose preference test. Tuley’s test; control, *n* = 28; resilient, *n* = 36; susceptible, *n* = 20 mice. **f** Radar graph showing changes in TF-activities in susceptible mice, normalized to levels in control mice. For each TF, the corrected mean ± sem of TF-activities are shown as log_2_-fold changes. See Supplemental Methods for details. **g** Heatmap illustrating the scaled transcripts per million (TPM) values of DEGs in the susceptible mice, compared to the same genes in control mice. RNA-seq analysis was performed on 3 mice from both the control and susceptible groups. **h** Scatter plot showing alterations in the transcriptional activity of each TF and the average change in expression of its downstream genes.

This study tests the hypothesis that sustained shifts in TF-activities drive recovery from depressive state and seeks to identify the specific TFs whose activity is persistently remodeled during this process. For this aim, we utilized TF-activity profiling [31, 32] to directly quantify their activity in mice subjected to repeated social defeat stress, a well-established rodent model of depression [5]. Such direct measurement of TF-activity is indispensable, because bulk mRNA profiles offer only an indirect, composite read-out of TF-activity, reflecting the synergistic actions of many regulators rather than the activity of any one TF [27–29]. Specifically, we generated TF-activity reporter mice via *in utero* viral infection at embryonic day 15 (Fig. 1a) [38]. This approach enables the targeted expression of TF-activity reporters, allowing for direct measurements of the precise activity of each TF in a specific population of neurons in vivo. At eight weeks of age, mice expressing the reporter constructs were subjected to a 10-day repeated social defeat stress (Fig. 1b), which resulted in behavioral tendencies toward depressive-like states, including social avoidance, anxiety-like behaviors, and anhedonia (Fig. 1c–e and Supplementary Fig. S1a). Social interaction tests revealed a sustained reduction in social interaction rate (SI-rate) in stressed mice, which gradually returned to control levels by 15 days after the end of the stress exposure (Supplementary Fig. S1b). Following the criteria established in previous studies, defeated mice were categorized as either resilient or susceptible according to their SI-rate [40] (Fig. 1c). Using these reporter mice, we evaluated the transcriptional activities of endogenous TFs as well as gene expression profiles in the anterior cortex, including the PFC, across resilient, susceptible and control groups. To identify TFs associated with the depressive phenotype, we employed a candidate-based approach [42]. We focused our analysis on a panel of 30 TFs previously implicated in stress responses, selected based on prior literature [21, 22, 32, 43, 44]. The quantified TF-activities were normalized to the mean values of the control group and integrated into a TFAP (Fig. 1f and Supplementary Fig. S1c; see Materials and Methods), revealing that multiple TFs exhibited altered activity patterns corresponding to the observed behavioral phenotypes.

To link the observed TF-activity alterations with their downstream transcriptional consequences, we performed RNA-seq on the identical brains from susceptible mice previously analyzed for TF-activity (Fig. 1a). This analysis identified 56 down-regulated and 67 up-regulated differentially expressed genes (DEGs; defined by *p* < 0.05 and fold-change (FC) > 2 or FC < 0.5) (Fig. 1g), with significant enrichment in several gene ontology (GO) terms (Supplementary Table S1). By integrating TFAP with transcriptomic data, we performed a multi-omics approach to identify core molecular hubs that regulate transcriptional alterations in susceptible mice. To this end, we compiled a list of TFs alongside their predicted downstream genes by matching TF binding motifs to the promoter regions of all genes (see Supplementary Methods). We then compared the transcriptional activity changes of each TF in the TFAP of susceptible mice with the corresponding average expression changes of their predicted downstream genes in the RNA-seq dataset (Fig. 1h). Through this multi-omics analysis, we identified TCF/LEF and REST as primary candidate TFs driving transcriptomic alterations associated with the depression-like state in mice. Both TCF/LEF and REST activity were higher in susceptible mice than in the resilient group (Supplementary Fig. S1c).

To evaluate whether these changes in the transcriptional activities of TCF/LEF and REST observed in mice are also relevant to humans with MDD, we analyzed published transcriptomic data from the postmortem cortex of MDD patients and matched control subjects [16] (Supplementary Table S2). As previously reported [16], a comprehensive analysis of gene expression patterns between patients with MDD and psychiatric controls revealed significant changes across multiple brain regions of the anterior cortex, including the anterior insula (aINS), ventromedial prefrontal cortex (vmPFC; BA25), orbitofrontal cortex (OFC; BA11), and dorsolateral prefrontal cortex (dlPFC; BA8/9), as well as in the nucleus accumbens (NAc), and ventral subiculum (vSub). Notably, we found that changes in gene expression were pronounced in genes downstream of either REST or TCF/LEF in multiple regions within them (REST, dlPFC, and NAc; TCF/LEF, aINS) (Supplementary Table S3).

Collectively, our multi-omics analysis, integrating direct TF-activity measurements with transcriptomic profiling of downstream genes, identified the potential involvement of specific TFs, TCF/LEF and REST, in the depressive phenotype in both mice and humans through the regulatory influence on gene expression. The heightened activity of TCF/LEF and REST observed in stress-susceptible mice after stress suggests that these transcriptional changes may contribute to the development of a depressive-like state or recovery during the post-stress period.

### TCF/LEF activity promotes recovery from a depressive state

**Fig. 2:**
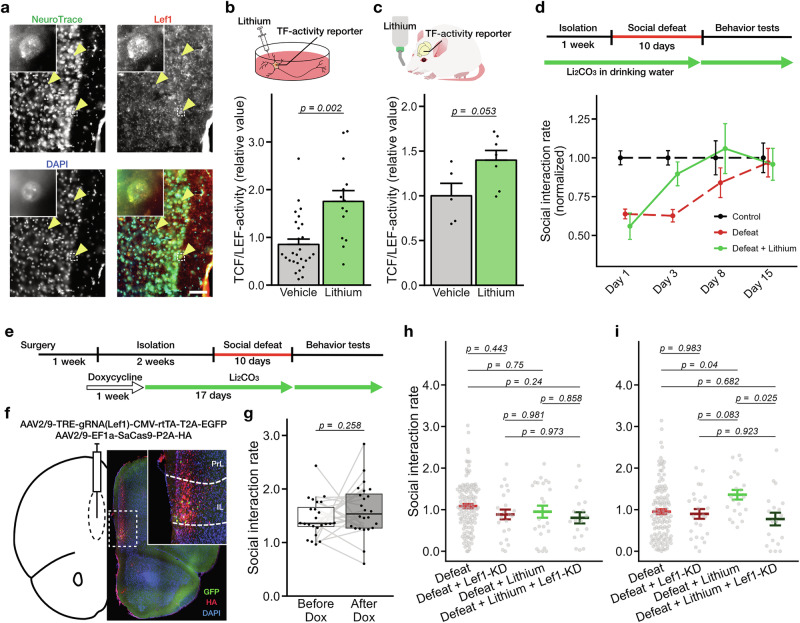
The role of TCF/LEF in the depressive phenotype. **a** An immunostained section of the anterior cortex. Scale bar, 100 μm. TCF/LEF-activity of cortical culture and the anterior cortex after lithium treatment. Mean ± sem; Welch’s *t*-test; vehicle, 27; lithium, 14 cultures in (**b**); vehicle, 5; lithium, 7 mice in (**c**). **d** SI-rate changes after defeat stress with lithium treatment. Experimental schedule (top). SI-rates normalized to the control group each day (bottom). The data include those shown in Fig. 1. The number of mice for control, defeat, and defeat + lithium—day 1: 83, 126, 23; day 3: 83, 126, 23; day 8: 25, 34, 19; day 15: 22, 29, 17. Social behaviors of *Lef1* knockdowned (Lef1-KD) mice. Scheme of the experiment (**e**). An example of the brain section (**f**). Scale bar; 500 μm. **g** SI-rates before and after doxycycline treatment without stress. Paired *t*-test, 24 mice. SI-rates of defeated mice with lithium treatment and Lef1-KD at day 1 **h** and day 3 (**i**) after the 10-day social defeat. Tukey’s multiple comparison test; defeat, 124 and 126; defeat + Lef1-KD, 23 and 24; defeat + lithium, 23 and 21; defeat + lithium + Lef1-KD, 18 and 19 mice in the post-stress days, respectively. Colored bars indicate mean ± sem.

Among the TF candidates identified, we first focused on the role of TCF/LEF, which exhibited a significant increase in activity in the susceptible mice (Dunnett’s test; *p* = 9.82 × 10^−3^ versus control, *n* = 14 and 10; Fig. 1f). TCF/LEF is well-known for its role in the Wnt signaling pathway, which regulates processes such as stem cell proliferation and differentiation [45, 46], however, its function in the adult brain is not well-characterized. Interestingly, Wnt-independent activation of gene transcription of TCF/LEF has been observed in neurons [47] and has been indicated to be linked to mechanisms of stress resilience in subcortical areas [48, 49]. Immunostaining of brain sections revealed that among the TFs of the TCF/LEF family, *Lef1* was expressed in cortical neurons, including those in the PFC of susceptible mice (Fig. 2a). Based on this observation, we hypothesized that the heightened TCF/LEF-activity observed in mice exposed to chronic stress plays a role in modulating the depressive state, and tested this by artificially activating TCF/LEF in the brain.

Lithium is known to inhibit GSK3β, thereby activating the gene transcription activity of TCF/LEF [50]. As expected, we detected an upregulation of TCF/LEF activity in cultured cortical neurons treated with lithium (50 mM for 24 h; Fig. 2b), as well as in the brains of mice subjected to chronic lithium administration (100 mg/kg/day for 17 days; Fig. 2c). Thus, we used lithium administration to pharmacologically achieve enhanced TCF/LEF activity, replicating the elevated activity observed in depressed mice. We observed that lithium treatment alone did not affect social interaction tendencies or behaviors in the open field in mice not subjected to chronic social stress (Supplementary Fig. S2a, b), nor have a significant effect on preventing depression phenotype following a 10-day social defeat stress (Fig. 2d, day 1; and Supplementary Fig. S2c). Intriguingly, however, we found that lithium exerted an antidepressant effect in individuals subjected to chronic stress. Their SI-rate measured on day 3 post-stress was significantly elevated and restored to levels comparable to those of the non-stressed control mice (Fig. 2d, days 3–15). The effect size, quantified using Hedge’s *g* on day 3 after the end of defeat stress, was 0.75. This anti-depressant effect of lithium depended on LEF1. Knockdown of endogenous *Lef1* expression in PFC had no observable impact on the scores of the social interaction test prior to stress exposure (Fig. 2e–g) but suppressed the effect of lithium to enhance post-stress recovery of the SI-rates (Fig. 2h, i, and Supplementary Fig. S3).

These results suggest that heightened TCF/LEF activity does not induce a depression-like state but rather promotes recovery from it. The increased TCF/LEF activity observed in susceptible mice may therefore reflect an ongoing process of recovery from a depressive state.

### REST activity is necessary for maintaining resilience to stress

**Fig. 3:**
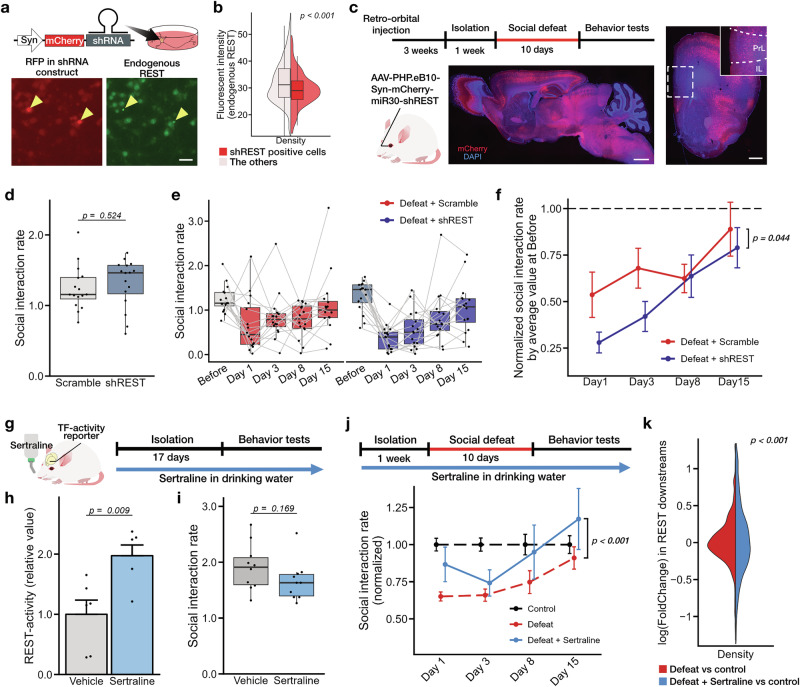
The role of REST in the depressive phenotype and sertraline-induced gene transcription in vivo*.* **a**, **b** Validation of shRNA targeting endogenous REST. Immunostained images of cultured cortical neurons. Scale bar, 50 μm. Welch’s *t*-test; shRNA positive, 140; others, 13,327 cells. **c**–**f** Effects of REST knockdown in vivo. **c** The experimental scheme (top) and the brain sections demonstrating the shRNA-expressing populations (bottom and right). The dotted area denotes the PFC; PrL, prelimbic; IL, infralimbic. Scale bar, 1000 and 500 μm, respectively. Changes in SI-rates in shRNA expressing mice. SI-rates before (**d**) and after social defeat (**e**), and SI-rates after chronic stress, normalized to the pre-stress values (**f**). Mean ± sem. Welch’s *t*-test and two-way ANOVA; shScramble, 16; shREST, 16 mice. Effects of sertraline in vivo. **g** The experimental schedule. Effect of sertraline on REST-activity in the anterior region of the cortex (**h**) and SI-rates (**i**) without social defeat stress. Welch’s *t*-test for (**h**) and (**i**). Number of mice: 6, 6 (**h**); 10, 10 (**i**). **j** Experimental schedule (top) and changes in SI-rates (bottom). Data include those shown in Fig. 1. SI-rates were normalized to the control group for each day. Mean ± sem; two-way ANOVA for defeat and defeat + sertraline. Number of mice for control, defeat, and defeat + sertraline — day 1: 90, 126, 21; day 3: 90, 126, 21; day 8: 27, 38, 15; day 15: 24, 33, 15. **k** Distribution of log_2_-fold changes in REST downstream gene expression; red, control versus defeat; blue, control versus defeat with sertraline treatment. *F*-test, 994 genes.

Next, we examined the role of the other candidate, REST. REST was originally identified as a transcriptional repressor, but it is now recognized to have dual functions, acting either to suppress or activate gene transcription, depending on context [51]. While the functions of REST have been predominantly studied in the context of neural development [52, 53], its involvement in psychophysiological disorders has also been documented [51, 54]. Given that REST is broadly expressed throughout the brain, we employed retro-orbital injection of viral vectors encoding short-hairpin RNA targeting *Rest* to achieve a widespread knockdown of endogenous REST (Fig. 3a–c). Among non-stressed subjects, repression of REST expression did not result in abnormalities in SI-rates (Fig. 3d) or anxiety-like behavior in the open field test (Supplementary Fig. S4a). However, following chronic stress, REST knockdown exacerbated the depressive phenotype of social avoidance (Fig. 3e, day 1–15). Specifically, when comparing SI-rates normalized to their respective pre-defeat levels, REST-knockdown mice exhibited significantly lower SI-rates following stress exposure compared to controls expressing scrambled shRNA (Fig. 3f). These results indicate that REST activity is required for maintaining resilience to stress.

**Fig. 4:**
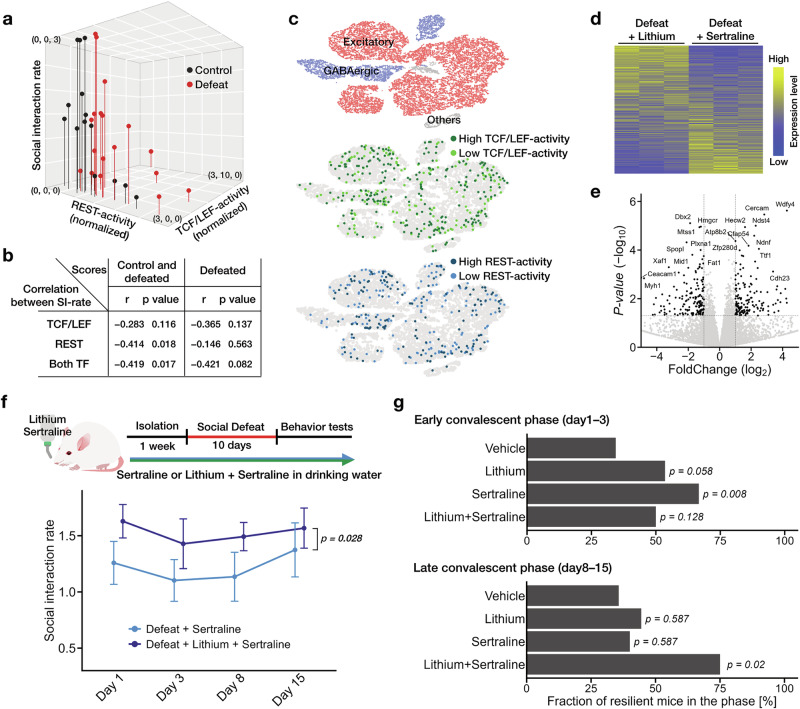
Cooperative role of TCF/LEF and REST in the recovery from depression. **a**, **b** SI-rate and TF-activities for each mouse expressing both TCF/LEF- and REST-reporters. 3D plot (**a**) and the Pearson correlation analysis (**b**). Control, 14; defeat, 18 mice. **c** Single-cell level TF-activity in PFC neurons. The *t*-SNE plot illustrates the clustered cells after social defeat (top) and the distribution of TF-activity for TCF/LEF (middle) and REST (bottom). See Fig. S6 for details. Each TF-activity was compared to the control average within the clusters, with cells showing high-activity and low-activity indicated in dark/light colors. **d**, **e** Results of bulk RNA-seq analysis in the anterior cortex of defeated mice treated with lithium or sertraline (*n*  =  3 mice each). TPM (**d**) and changes between the two drug treatments (15,881 genes) (**e**). The dotted lines indicate *p*-value of 0.05 and log_2_-fold changes of ± 1. **f** Effects of multiple drug treatments. Experimental scheme (top) and changes in SI-rates. Mean ± sem; two-way ANOVA; number of mice in each cohort — sertraline and lithium + sertraline; day 1: 14, 12; day 3: 15, 12; day 8: 15, 12; day 15: 15, 12. **g** The proportion of socially defeated mice not exhibiting depressive symptoms following drug treatments, compared between days 1–3 (top) and days 8–15 (bottom) post-stress exposure. The *p*-values were calculated based on a binomial test compared to vehicle-treated controls and corrected using the Holm method. Summarized data include those shown in Figs. 2d, 3j, 4f and Supplementary Fig. S1b.

To test the hypothesis that REST activity promotes stress resilience, we next focused on pharmacological approaches to enhance REST activity in vivo. A recent study has shown that sertraline, a widely prescribed antidepressant, binds to the nuclear protein mSin3 and modulates its interaction with REST, a complex involved in the regulation of gene transcription [55]. Therefore, we assessed the impacts of sertraline on REST-mediated gene regulation and mice behaviors. Analysis with a REST-activity reporter revealed upregulation of the transcriptional activity of endogenous REST following the administration of sertraline (5 mg/kg/day for 17 days) (Fig. 3g, h). The upregulation of REST-activity was also observed in cortical culture in vitro, which is devoid of serotonergic neurons, indicating that the activation occurs independent of serotonin [56] (Supplementary Fig. S5a–e). Administration of sertraline to non-stressed mice had no effect on SI-rates (Fig. 3i) or open-field behavior (Supplementary Fig. S4b), indicating that activation of REST under baseline conditions does not alter depression-related behaviors. However, when mice were subjected to chronic social defeat, sertraline treatment significantly mitigated the decline in sociality (Fig. 3j). The effect sizes, quantified using Hedge’s *g*, were 0.60, 0.18, 0.36, and 0.45 at 1, 3, 8, and 15 days post-social defeat, respectively. Sertraline treatment also affected anxiety-related behaviors after stress, as evidenced by increased time spent in the center zone of the open field, confirming its effectiveness as an antidepressant (Supplementary Fig. S4c). Transcriptome analysis of the anterior cortex revealed that sertraline treatment induced more pronounced fluctuation in the expression of REST downstream genes that were differentially expressed in mice subjected to social defeat (Fig. 4k). Since the transcriptomic change observed in vivo may include gene expression alterations induced by sertraline’s influence on serotonin pathways, as well as indirect effects mediated downstream of REST-regulated transcription, we assessed the transcriptomic change induced by sertraline in cortical neuron culture (Supplementary Fig. S5d–f). This analysis revealed that sertraline caused alterations in the gene expression profile compared to vehicle-treated culture, with REST-regulons being more significantly affected than non-REST downstream genes (Supplementary Fig. S5f). Further Gene Set Enrichment Analysis (GSEA) in GO terms revealed that the upregulated gene list was enriched in multiple pathways related to cell-signaling and neural plasticity, while the downregulated gene list prominently featured pathways related to immune function and apoptosis (Supplementary Table S4). Collectively, these findings suggest that the gene transcriptional activity of REST is associated with stress resilience and recovery from a depressive-like state.

### Cooperative role of TCF/LEF and REST in the recovery from a depressive state

We identified the involvement of the two TFs, TCF/LEF and REST, in mice exhibiting a depressive phenotype. Given that the drugs affecting either TCF/LEF or REST influenced depressive behaviors, we next examined their potential synergistic effects in promoting recovery from the depression-like state. Firstly, we simultaneously measured TCF/LEF and REST activity in individual mice subjected to social defeat stress. We noticed that the activities of both TCF/LEF and REST tended to exhibit an anti-parallel correlation with their SI-rate in both the control and defeated groups (Fig. 4a). Moreover, the correlation coefficient between SI-rate and TF-activity in defeated mice was weaker when TCF/LEF and REST were analyzed jointly, compared to when each TF was considered individually (Fig. 4b), suggesting that both TCF/LEF and REST contribute individually to the depressive phenotype. Second, to reveal the cellular-level involvement of TCF/LEF- and REST, we performed TF-activity measurements and transcriptome analysis using single-nucleus RNA-seq in mice expressing both TCF/LEF- and REST-reporter viruses (Fig. 4c; Supplementary Fig. S6a, b; and see Supplemental Methods). Stress-induced changes in the activity of both TFs were found to be heterogeneous at the single-cell level, with elevated activity not restricted to specific cell populations (Fig. 4c and Supplementary Fig. S6c, d). For both TCF/LEF and REST, we observed stress-induced changes in their activity across all layers of the PFC, indicating that these changes occur broadly within the brain (Fig. 4c and Supplementary Fig. S6e). Third, transcriptome analysis of the anterior cortex in defeated mice treated with either lithium or sertraline revealed that the gene expression changes induced by the two drugs were largely distinct (Fig. 4d, e). Together, these findings suggest that both TCF/LEF and REST contribute to stress-induced cellular changes and may independently influence recovery from a depressive state.

In human patients with depression, multiple antidepressants are occasionally used in combination when a primary drug fails to produce satisfactory therapeutic outcomes [4, 57, 58]. Lithium is one such drug that is used for antidepressant augmentation [11, 58–60]. The aforementioned observation at both cellular and individual levels implies that the combined drug treatment targeting TCF/LEF and REST increases the likelihood of having a therapeutic effect. To test this hypothesis, we administered sertraline and lithium to mice either in combination or separately and assessed their impact on the behavioral phenotype after chronic social defeat stress. We observed that the mice treated with both sertraline and lithium together showed an increased SI-rate compared to the mice treated with sertraline alone (Fig. 4f). Assessment of depressive state changes in each mouse revealed that both lithium and sertraline exhibit therapeutic effects in the early week after the stressful experience (Fig. 4g; days 1–3). However, greater improvement was observed in the later week with co-administration of both drugs, compared to the administration of single drugs (Fig. 4g; days 8–15). Collectively, these results imply that the behavioral phenotype related to chronic stress undergoes alterations due to the changes in the activity of various TFs, including TCF/LEF and REST, with the extent of influence differing among individuals; thus, applying a combination of pharmacological agents is likely to augment the therapeutic effects against depression.

## Discussion

Chronic stress causes widespread transcriptional remodeling in the brain, perturbing numerous biological pathways and ultimately impairing neural function. Conversely, recovery necessitates an equally extensive reconfiguration of these networks to re-establish homeostasis. Pinpointing the master regulators that orchestrate these bidirectional state transitions remains challenging because multiple converging factors act synergistically and confound straightforward causal attribution. In this study, we specifically focused on the upstream molecular mechanisms regulating these intricate gene expression patterns. We employed a method for directly measuring TF-activities in vivo and identified TCF/LEF and REST as critical regulators in the recovery phase of a depression-like state, both exhibiting elevated activity in their brains. Furthermore, our findings reveal that lithium and sertraline, two neuropsychiatric drugs already in clinical use, modulate the gene transcriptional activities of TCF/LEF and REST. These results highlight that dysregulation of gene expression mechanisms is not only a cause of the pathophysiology of depression but also underlies the therapeutic efficacy of antidepressants. Furthermore, this suggests that aside from their primary pharmacological actions, these agents may influence the gene regulatory functions of TCF/LEF and REST, thereby impacting therapeutic outcomes.

The pathophysiology of depression remains incompletely understood, largely due to the heterogeneity of its underlying mechanisms [5, 19, 20]. Our data, which revealed individual variability in TCF/LEF and REST activity within the brains of chronically stressed mice, align with the notion of depression as a heterogeneous disorder. Additionally, the observed heterogeneity of TCF/LEF and REST activity at a cellular level also emphasizes the complexity of depression, in which multiple molecular mechanisms, including these transcription factors, contribute to both the etiology and recovery process of depression, even within a single subject. The discovery of the distinct pathways, TCF/LEF and REST, involved in the recovery mechanism, along with their heterogeneous contributions among the subjects, may help explain clinical observations that switching or augmenting antidepressant medications enhances treatment success [57–60]. The observed heterogeneity in the molecular mechanisms associated with depression and recovery supports the view that personalized approaches—targeting the specific pathways most relevant to each patient’s symptoms—may be ideal for optimizing therapeutic strategies and improving treatment efficacy in depression [61, 62]. The TFAP analysis further suggests the potential involvement of additional TFs beyond TCF/LEF and REST, in establishing or recovering from the depressive phenotype. We also acknowledge that TFs not included in our TFAP analysis could also contribute to the phenotype. In addition, while we identified TFs that appear to naturally promote recovery from the depressive state, and offered potential targets for pharmacological intervention, the upstream mechanisms driving changes in TF-activity in individual stressed animals remain unclear. These considerations underscore the need for further investigation to identify additional key molecules beyond TCF/LEF and REST, as well as the mechanisms underlying their modulation. Another major limitation of this study not addressed is sex differences. Our study focused exclusively on male mice for stress-induced depression-like state, as the chronic stress paradigm used to induce depressive phenotypes is established and has been evaluated only in males of this strain. Further studies are necessary to address whether the findings are relevant for female mice.

While recent neuroscience research has prioritized the identification of specific neural circuits linked to distinct behaviors and disease symptoms, it is increasingly recognized that the etiology of many neuropsychiatric disorders may stem from the dysfunction of multiple dispersed neural circuits [5, 7, 63, 64]. Consistent with this view, our findings demonstrated that alterations in TCF/LEF and REST activity occur broadly across neural populations within affected subjects. Moreover, pharmacological interventions often induce broad, systemic effects throughout the brain, rather than selectively targeting specific cellular populations [65]. Thus, establishing more practical approaches to pharmacological interventions for depression requires an understanding of the dynamic molecular changes across the entire brain. The methodology used in this research not only quantifies the gene transcriptional activity of each TF but also evaluates gene expression profiles in depressed brains, offering a detailed visualization of the molecular landscape and identifying the candidate targets for therapeutic intervention. Such efforts will pave the way for research aiming at deciphering the molecular origins of chronic disorders and facilitate the development of preventative or therapeutic treatments for neurological diseases. Further studies, encompassing the acquisition of TFAPs from various neuropathological models and the evaluation of medication effects on these profiles, will substantially contribute to establishing more efficient therapeutics.

## Supplementary information

Supplementary Material

## Data Availability

The data that support the findings of this study are available upon reasonable request from the corresponding author. All sequencing data are available from the DDBJ BioProject database with accession number PRJDB17042.
