# Supplementary material for: Transcription factors Lef1 and Rest stimulate recovery from depressive states
Source: Neuropsychopharmacology. 2025 Oct 7;51(5):846–55. doi: 10.1038/s41386-025-02259-0 (PMC13013837; doi:10.1038/s41386-025-02259-0)
Supplement: Supplementary file 1 — Supplementary Material [file 41386_2025_2259_MOESM1_ESM.pdf]

Supplemental Material

Transcription factors Lef1 and Rest stimulate recovery from depressive states

Hajime Yamamoto, Satomi Araki, Ryoma Onodera, Yasuhiro Go, and Kentaro Abe

Table of contents

Supplementary Methods

Supplementary Figure S1 to S6

Supplementary Table S1 to S4

Supplementary References

Supplementary Methods

Cell cultures

Cortical neurons were obtained from mice (Slc: ICR) embryos at embryonic day 15 (E15). The cortex was dissected and treated with 0.05 % trypsin (Fujifilm-Wako) at 37°C following mechanical dissociation to yield single cells and then plated onto poly-L-lysine (SIGMA, #P2336) coated tissue-culture plates (IWAKI, #3820-024). The cultured neurons were maintained in Neurobasal Medium (Thermo Fisher, #21103049) supplemented with B27 Plus supplement (1:50; Thermo Fisher, #A3582801), Gultamax-I (1:100; Thermo Fisher, #35050061), and penicillin-streptomycin (1:100, Fujifilm-Wako) at 37°C under 5% CO_2. Those neurons were transfected with viral vectors at 3 days *in vitro* (div). To verify knockdown of Lef1, we used thalamic neuron culture, which expresses Lef1 in most of the neurons (Supplementary Fig. S3). Thalamic culture was prepared from E12 mouse embryos and processed similarly to the cortical culture described above.

RT-PCR analysis

The total RNA collected from the cortical culture at 14 div and surgically dissected samples, including raphe nucleus, were reverse-transcribed using Revatra-ace qPCR RT Master-mix with gDNA remover (Toyobo) according to the manufacturer's protocol. The resultant cDNA was amplified using PrimeSTAR (Takara) with the following cycling conditions: 95°C for 10 s, 63°C for 20 s, and 72°C for 15 s, on a PCR machine (T100, Biorad). PCR primer pairs used were 5'-ACACCCCGGAACCAGATACAT-3' and 5'-TTCCTGCAAGCAGGTCGTCT-3' for Tph2, and 5'-GGAGCCAAGTCCGTTGTCCT-3' and 5'-GCCGGCTCAGCTTTAACCTTG3' for Pgk1. The amplified DNA products were electrophoresed on an agarose gel (Nippon-gene), stained with UltraPower DNA/RNA safe dye (Gellex), and photographed by a gel imaging device (Stage-2000, AMZ systems science).

Transcriptome and TF binding site enrichment analysis

The same RNA samples utilized for measuring the TF-activity were used in the transcriptomic analysis of bulk RNA-seq. The samples from the anterior cortex of control and stressed mice exhibiting social avoidance underwent mRNA purification, poly-A mRNA enrichment library preparation, and sequencing on Illumina NovaSeq-6000 platform (150 bp paired-end), performed by Novogene. For the analysis of bulk RNA-seq data, raw reads underwent quality control using Fastp (v0.23.4) and were then aligned to the reference mouse genome (GRCm39) with Salmon (v1.8.0). Subsequently, we performed differential gene expression analysis with tximport (v1.30.0) and DESeq2 (v1.36.0) packages from Bioconductor in R. Gene set enrichment analysis with the Benjamini-Hochberg procedure was performed with clusterProfiler package (v4.10.1) in R, focusing on Molecular Function (MF) in the analysis on cultured neurons. We evaluated TFBS enrichment on the sequence of the promoter region (± 1,000 bp from TSS), which information was obtained from UCSC Genome Browser on Mouse (GRCm39) with JASPAR package (v0.99.7) using TFBStools package (v1.34.0) in R. The JASPAR Matrix IDs used in TF motif analysis in transcriptome data of mouse data are the followings: AR, MA0007.3; ATF6, MA1466.1; C/EBP, MA0102.2; CREB, MA0018.2; EGR1,

MA0162.1; FOXO, MA0480.2; Gli1, MA1990.1; GR, MA0113.2; gre, MA0113.2; MEF2, MA0660.1; MR, MA0727.1; MYC, MA0104.3; NFAT, MA0624.2; P53v2, MA0106.3; RAR, MA0729.1; RBP, MA1621.1; RELB, MA0107.1; REST, MA0138.2; SMAD1/5, MA1557.1; SMAD2/3/4, MA1153.1; SP1, MA0079.2; SREBF, MA0829.1; SRF, MA0083.3; STAT1/2, MA1623.1; TCF/LEF, MA0769.1; and tre, MA1535.1. TF downstream genes were defined as the 1,000 genes with the highest relScore, one of the parameters reflecting similarity to the consensus sequence. The sequence data obtained in this study were deposited in the DDBJ BioProject database with accession number PRJDB17042.

For the transcriptome analysis of human postmortem tissue, we utilized published RNA-seq data (Gene Expression Omnibus (GEO) database; GSE102556), obtained from the specimen at the Douglas Bell Canada Brain Bank (DBCBQ; Douglas Mental Health Institute, Verdun, Québec) (1). This dataset includes data from both male and female subjects. We utilized the data from both sexes, encompassing all brain regions in this record, including the orbitofrontal cortex (OFC; BA11), dorsolateral prefrontal cortex (dlPFC; BA8/9), ventromedial prefrontal cortex (vmPFC; cingulate gyrus 25, BA25), anterior insula (aINS), nucleus accumbens (NAc), and subiculum (Sub). For the raw read data, we sequentially performed a quality control with fastp (ver. 0.23.4), alignment to the human genome GRCh38 with STAR (ver. 2.7.11a), and gene expression quantification with RSEM (ver. 1.3.1). Samples with markedly fewer reads mapped uniquely were removed from further analysis. Upstream sequences of each gene were retrieved from the UCSC Genome Browser for the human genome (GRCh38). Gene downstream of TCF/LEF and REST were identified using JASPAR2022, applying the same parameters as those used in the mouse experiments. The JASPAR Matrix IDs used in TF motif analysis in transcriptome data of human data are REST, MA0138.2 and TCF/LEF, MA1421.1.

<u>Single nucleus RNA-seq analysis</u>

The LV-based TF activity reporters cannot be utilized for RNA-seq analysis because the mRNAs of its reporters and references lack poly-adenyl (pA) tails and thus can not be read by oligo-dT mediated sequence library preparation methods. Therefore, we transferred these constructs to AAV to allow the addition of pA tails to the references and reporters for single-cell RNA-sequence. To create such constructs, we cloned the LV-based reporter constructs into AAV vectors, allowing bi-promoter expression of the reporter gene and reference gene by a single vector. To reduce the size of constructs, we cloned only the N-terminal region (234 bp, srtRep#5) (2) of reporter Rep#5 and affixed to its 3' region a unique molecular identifier (UMI) sequence (5'-CATGTTGA-3') and a bovine growth hormone polyadenylation signal (bGHpA) sequence (5'-CTGTGCCTTCTAGTTGCCAGCCATCTGTTGTTTGCCCCTCCCCCGTGCCTTCCTTG ACCCTGGAAGGTGCCACTCCCACTGTCCTTTCCTAATAAAATGAGGAAATTGCAT CGCATTGTCTGAGTAGGTGTCATTCTATTCTGGGGGGTGGGGTGGGGCAGGACAG CAAGGGGGAGGATTGGGAAGAGAATAGCAGGCATTGGGGA-3'). These constructs were replaced to original reporter gene of LV-REST/NRSF-reporter (reporter gene, Rep#5; reference gene, Ref#5) (2). In addition, the reference gene, Ref#5, was replaced with a tagRFP gene affixed with a UMI sequence (5'-CTGCAGTA-3') and an SV40 early polyadenylation

signal sequence (5’-TCGGTCCAGTGAAAAAAATGCTTTATTTGTGAAATTTGTGATGCTATTGCTTTATTTGTAACCATTATAAGCTGCAATAAATCGAACTAGTATC-3’) to aid a visual dissection of transfected cells under fluorescent microscopes. This resulted in pAAV-REST-srtRef#5-Pgk-tagRFP. Similarly, AAV vector for TCF/LEF reporter was created from LV-TCF/LEF-reporter (reporter gene, Rep#3; reference gene, Ref#3) (2). The reference gene was replaced with mCherry gene affixed with UMI sequence (5’-GACTCTAT-3’) and an SV40pA sequence, and the reporter gene was replaced by N-terminal region of Rep#3 (102 bp, srtRep#3) affixed with UMI sequence (5’-CCGTATAT-3’) and bGHpA sequence, resulting in pAAV-TCF/LEF-srtRep#3-Pgk-mCherry. Plasmid used for creating AAVs for single-cell TF-activity measurement will be distributed through Addgene. For single-cell TF-reporter analysis, we injected AAV-based TF-activity reporters (AAV2/9-TCF/LEF-srtRep#3-Pgk-mCherry, AAV2/9-REST-srtRep#5-Pgk-tagRFP) into the PFC of 8 weeks male mice. After recovery, these mice were subjected to chronic social defeat stress. The brains of three non-stressed control and three mice showing depressive phenotype after repeated social defeat stress were dissected, and frontal slices (~ 1 mm thick) were created. Under a fluorescence stereomicroscope, the PFC, where red-fluorescent signals from reference gene (mCherry and tagRFP) were observed, was dissected out (~ 1 mm^3) and stored frozen at −80 °C. From those frozen specimens, nuclei were isolated using a nuclei isolation kit (Minute Detergent-Free Nuclei Isolation Kit, Invent Biotechnologies, Inc., #NI-024) and collected DAPI-positive nuclei by a cell sorter (SH800, Sony). Collected nuclei were subjected to single-cell library preparation using Chromium NEXT GEM Single Cell 3’ HT Library Kit v3.1 (10× Genomics, #PN-1000370). The constructed libraries were sequenced on MGI DNBSEQ-T7 (150 bp paired-end) platform. Raw reads (about 1.2–1.6 billion in the control and defeated samples) were analyzed using Cell Ranger (v7.1.0, 10× Genomics). In concrete, resultant FASTQ files were aligned to the mouse genome (GRCm38) from 10× Genomics (https://support.10xgenomics.com/single-cell-gene-expression/software/release-notes/build) and to the specific sequence located in TF-activity-reporter constructs. Cell cluster analysis was performed with Seurat package (v5.0.3) in R. After quality control with ddqcR package (v0.1.0) and doublet removal with DoubletFinder package (v2.0.4), the data of remaining cells in the control and defeated mice was normalized and integrated by IntegrateLayers function in Seurat. Following the data integration, we conducted dimensionality reduction, neighbor searching, and clustering. Subsequently, the data was visualized as a *t*-SNE plot. The expression of known marker genes was used to assign identities for each cluster: *Snap25* for neurons, *Slc17a7* for excitatory neurons, *Gad2* for GABAergic neurons, *Calb1*, *Cux1*, and *Cux2* for layer 2/3, *Rorb* for layer 4, *Fezf2* and *Bcl11b* for layer 5, and *Tle4*, *Syt6*, and *Foxp2* for layer 6 of the cortex. After clustering cells based on their transcriptome, we categorized cells that exhibited a positive difference in the reporter-to-reference expression ratio in the defeated mice groups, compared to that ratio in the corresponding cluster of the control group as having “high activity.” These data were deposited in the DDBJ BioProject database with accession number PRJDB17042.

Supplementary Figures

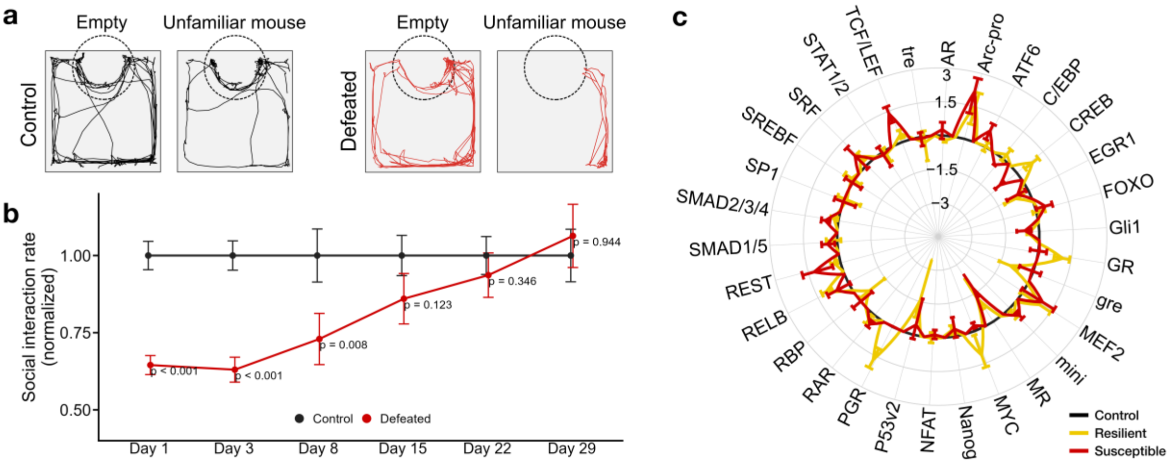

Supplementary Figure S1. Results of social interaction tests and TF-activity profiling in the mice subjected to chronic social defeat stress.

(**a**) An example of trajectories in the social interaction test. The dotted line shows the interaction zone where an experimental mouse can interact with an unfamiliar mouse. (**b**) Social interaction (SI)-rate change of controls and defeated mice after repeated social defeat stress. The SI-rates for each day were normalized to the corresponding control group. Mean ± sem; repeated two-way ANOVA, post-hoc Tukey's test; mice per cohort: day 1, n = 79, 122; day 3, n = 79, 122; day 8, n = 21, 34; day 15, n = 18, 29; day 22, n = 15, 16; day 29, n = 14, 16. (**c**) Radar graph representing the TFAPs of mice classified as resilient or susceptible. For each TF, the corrected mean ± sem of TF-activities are shown as $\log_2$-fold change relative to control levels. See Material and Methods for details. Data include those shown in Fig. 1f.

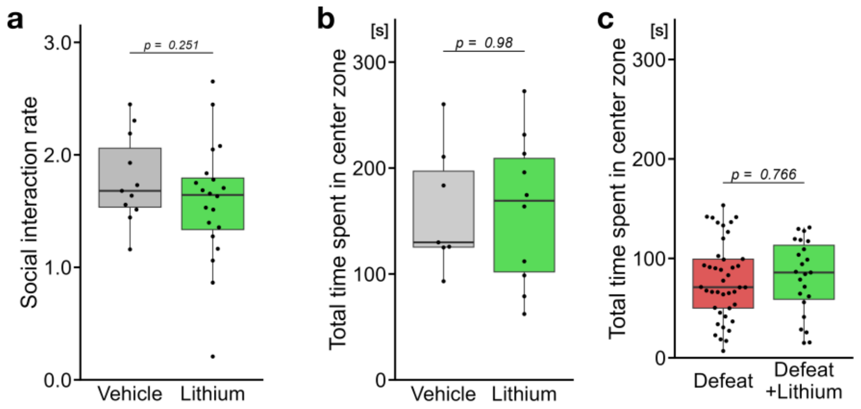

Supplementary Figure S2. Effects of lithium administration on mouse behavior.

(**a**, **b**) Behaviors of lithium-treated mice without defeat stress. (**a**) SI-rate of the mice following 17 days of lithium treatment (vehicle, 11; lithium, 20 mice). (**b**) Total time spent in the center zone for those mice (vehicle, 7; lithium, 10 mice). (**c**) same as (**b**) in defeated mice with or without lithium treatment (defeat, 42; defeat + lithium, 23 mice). Welch's *t*-test was used for the statistical analyses shown in these figures.

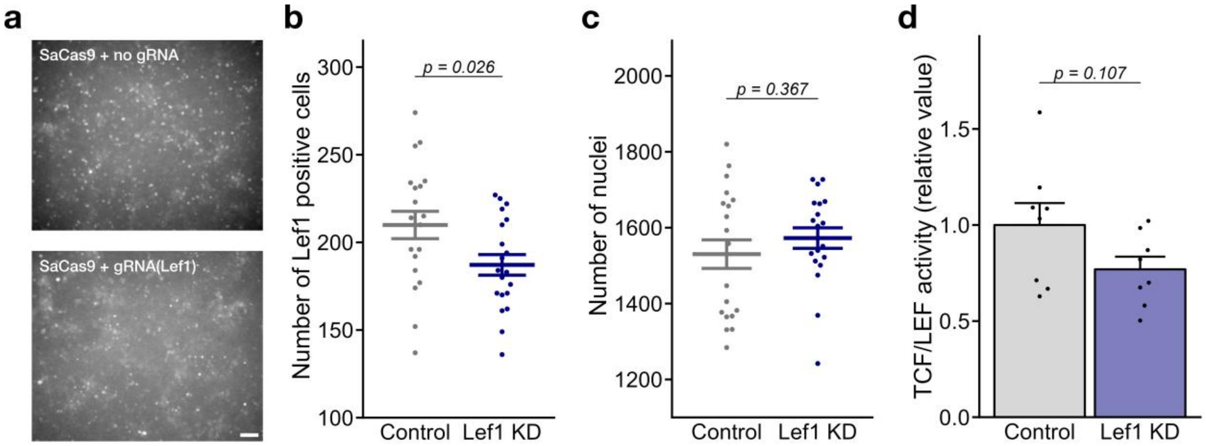

Supplementary Figure S3. Validation of *Lef1* knockdown

(**a**) Images of thalamic neuron culture, immunostained with anti-LEF1 antibody. The cultures were transfected with AAV to express SaCas9, either without (top) or with gRNAs targeting for *Lef1* (bottom), and treated with lithium for 24 hours. Scale bar; 100 µm. (**b**, **c**) The number of cells per 50 mm^2 positive for LEF1 (**b**) or DAPI (**c**) with or without the gRNA (control, 20; Lef1 KD, 20 cultures). Mean ± sem, Welch's *t*-test. (**d**) TCF/LEF-activity of Lef1-KD cells measured by TCF/LEF-activity reporter viruses (control, 8; Lef1 KD, 8 cultures); Mean ± sem, Welch's *t*-test.

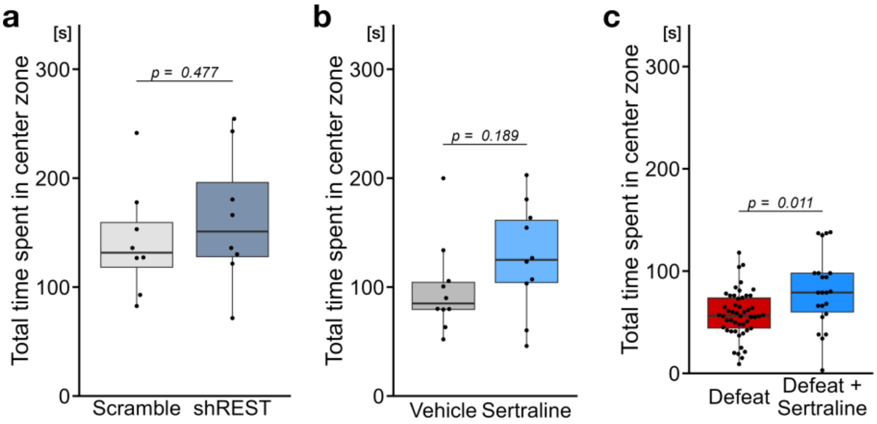

Supplementary Figure S4. Effect of REST knockdown and sertraline on anxiety-like behaviors.

(**a**–**c**) Total time spent in the center zone in the open field test. (**a**) A result from mice expressing shREST (scramble, 8; shREST, 8). (**b**, **c**) Results from mice treated with sertraline (vehicle, 10; sertraline, 10; defeat, 20; defeat + sertraline, 22 mice). Welch's *t*-test was used for the statistical analyses shown in these figures.

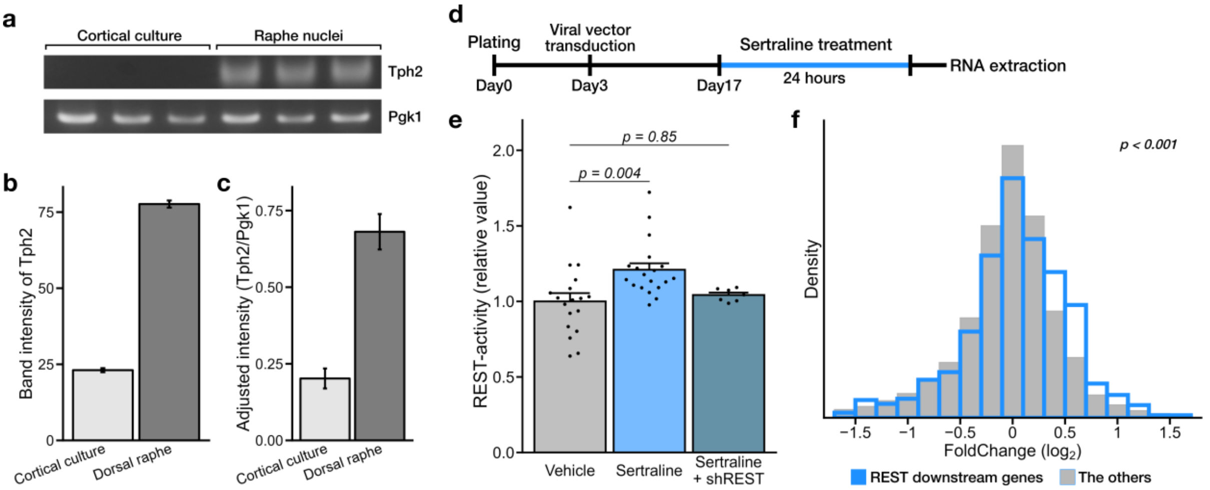

Supplementary Figure S5. Serotonin-independent transcriptomic alterations induced by sertraline via REST regulation

(**a**) Image of gel electrophoresis showing the lack of expression of endogenous *Tph2* in cultured cortical neurons. The expression of *Tph2*, a marker for serotoninergic neurons, and *Pgk1*, a housekeeping gene, were analyzed by reverse transcription PCR (RT-PCR). (**b**, **c**) Quantification of PCR bands; mean ± sem, $n = 3$ mice or cultures. (**d**–**f**) Evaluation of REST-activity and transcriptome in cortical cultures, measured using the REST-reporter. (**d**) Scheme of the experiment. (**e**) Comparison of REST-activities in cortical cultures treated with or without sertraline, and with or without shREST. Mean ± sem, Dunnett's *t*-test; vehicle, 18; sertraline, 19; sertraline with shREST, 7 cultures. (**f**) Histogram showing $\log_2$-fold changes in the expression of REST downstream genes compared to other genes in sertraline treated cultures. Wilcoxon signed-rank test; REST downstream and other genes, 997 and 14,590.

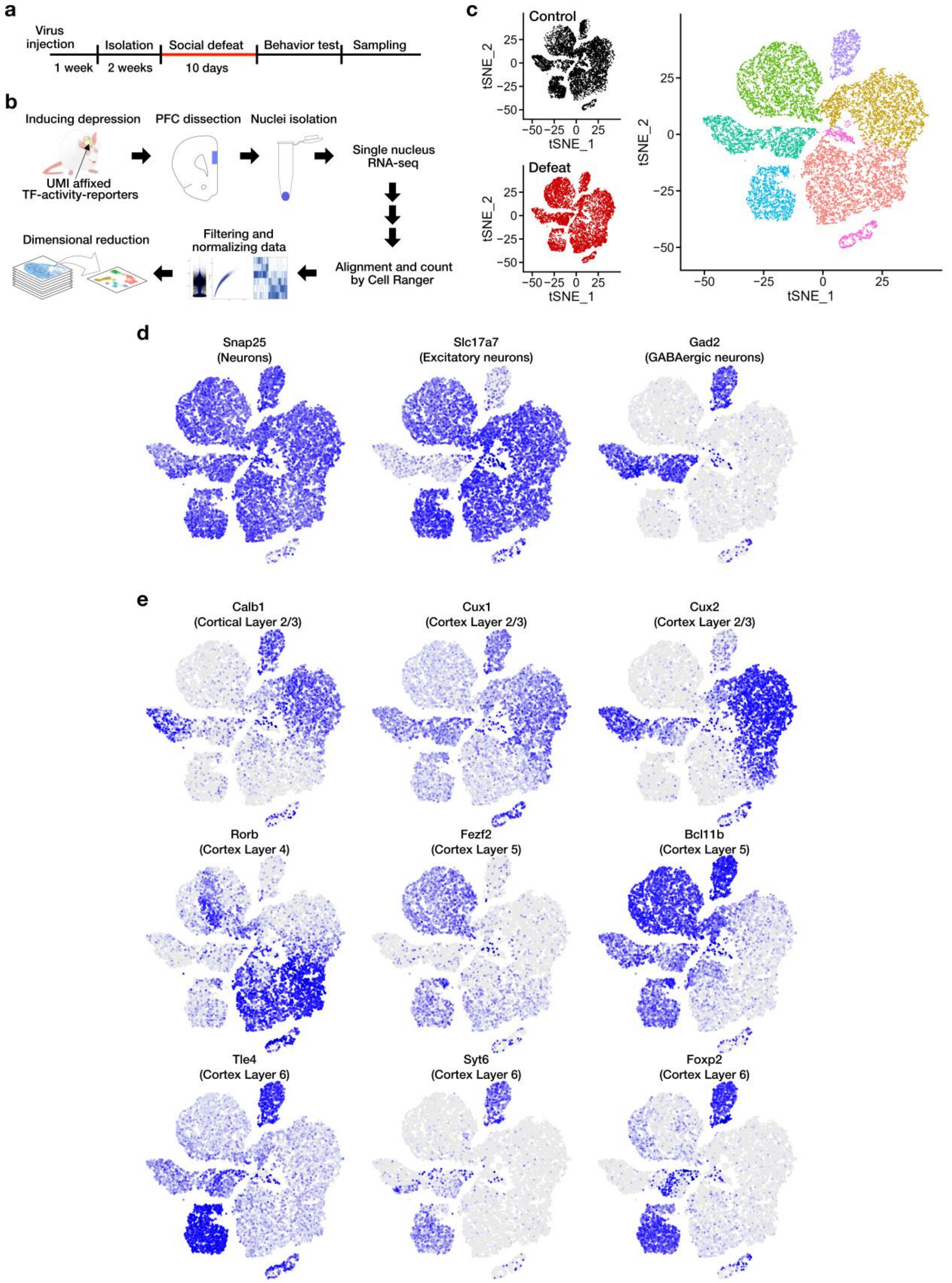

Supplementary Figure S6. Single-cell transcriptome analysis.

(**a**, **b**) The experimental scheme used for single-nucleus RNA-sequencing (**a**) and its procedure (**b**). Mice were injected with TF-reporters for TCF/LEF and REST in their PFC. (**c**) The *t*-SNE plots depicting single-cell transcriptome data from the control (12,180 cells)

and defeated mice (12,618 cells) (left), with plots colored according to three individual clusters (right) as shown in Figure 4c. (**d**, **e**) The expression of cell-type or region-specific marker genes, with cells exhibiting high expression, is highlighted in blue.

Supplementary Tables

Supplementary Table S1. Enriched GO terms in gene expression profiles of the brains of mice subjected to chronic social defeat.

Description	ID	NES	pvalue	p.adjust
Positive				
G protein-coupled receptor activity	GO:0004930	1.873	5.73×10^{-8}	4.59×10^{-4}
Negative				
Negative regulation of cell junction assembly	GO:1901889	−2.221	2.41×10^{-5}	2.15×10^{-2}
Microtubule bundle formation	GO:0001578	−1.996	2.37×10^{-5}	2.15×10^{-2}
Axoneme assembly	GO:0035082	−1.992	5.78×10^{-5}	4.21×10^{-2}
Ciliary plasm	GO:0097014	−1.968	2.66×10^{-6}	9.08×10^{-3}
Axoneme	GO:0005930	−1.966	4.54×10^{-6}	9.08×10^{-3}
Cytoplasmic translation	GO:0002181	−1.943	1.79×10^{-5}	2.15×10^{-2}
Structural constituent of ribosome	GO:0003735	−1.941	1.27×10^{-5}	2.04×10^{-2}
Ribosomal subunit	GO:0044391	−1.931	4.09×10^{-6}	9.08×10^{-3}
Cilium movement	GO:0003341	−1.811	6.99×10^{-5}	4.66×10^{-2}

Supplementary Table S2: Summary of sample data used for the analysis of human postmortem brain tissues.

Tissue	Phenotype	Gender	Age	Number of Cohorts
aINS	CTRL	Female	58.1 ± 19.5	9
aINS	CTRL	Male	41.2 ± 11.3	13
aINS	MDD	Female	43.7 ± 11.6	13
aINS	MDD	Male	46.7 ± 15.7	13
vmPFC	CTRL	Female	57.1 ± 19.9	7
vmPFC	CTRL	Male	40.5 ± 13.3	8
vmPFC	MDD	Female	44.2 ± 11.2	10
vmPFC	MDD	Male	59.3 ± 10	3
dlPFC	CTRL	Female	58.1 ± 19.5	9
dlPFC	CTRL	Male	41.2 ± 11.3	13
dlPFC	MDD	Female	43.7 ± 11.6	13
dlPFC	MDD	Male	46.7 ± 15.7	13
OFC	CTRL	Female	58.1 ± 19.5	9
OFC	CTRL	Male	41.2 ± 11.3	13
OFC	MDD	Female	45.2 ± 10.6	12
OFC	MDD	Male	46.7 ± 15.7	13
NAc	CTRL	Female	58.1 ± 19.5	9
NAc	CTRL	Male	41.2 ± 11.3	13
NAc	MDD	Female	43.7 ± 11.6	13
NAc	MDD	Male	47.3 ± 14.6	15
vSUB	CTRL	Female	51.7 ± 17	7
vSUB	CTRL	Male	40.8 ± 11.7	12
vSUB	MDD	Female	45.2 ± 10.6	12
vSUB	MDD	Male	48.2 ± 15.3	12

Supplementary Table S3. Results from the transcriptome analysis regarding REST and TCF/LEF downstream genes in human MDD patients.

Tissue	REST downstream			The others			pvalue
	Median	Skewness	Kurtosis	Median	Skewness	Kurtosis	
aINS	0.015415327	0.7838719	5.097120	0.015803764	0.1384594	10.26625	9.07×10^{-1}
vmPFC	0.005800326	-0.4819545	9.472586	0.015563984	0.2152455	12.98003	1.29×10^{-1}
OFC	-0.003755119	-0.7137906	9.169242	-0.003123652	0.0814217	13.36203	6.30×10^{-1}
dlPFC	0.002825973	-0.4119188	10.840184	0.013060088	-0.2952381	10.35302	8.08×10^{-3}
vSUB	-0.003433500	-0.4108836	7.350923	-0.011123804	0.1348232	11.02118	9.38×10^{-2}
NAc	-0.007761860	-0.1232795	9.174909	-0.022028087	-0.3159321	12.59076	1.93×10^{-5}

Tissue	TCF/LEF downstream			The others			pvalue
	Median	Skewness	Kurtosis	Median	Skewness	Kurtosis	
aINS	0.020386884	0.17243409	7.268092	0.004977369	0.15330063	10.64400	4.44×10^{-16}
vmPFC	0.007119217	0.05445320	9.250995	0.003782382	0.24962516	13.47372	1.52×10^{-1}
OFC	-0.005850189	0.18680350	6.913177	-0.001095924	0.03079249	14.90741	1.40×10^{-3}
dlPFC	0.003446640	-0.47341351	8.958947	-0.0005143638	-0.24052683	10.53795	5.51×10^{-2}
vSUB	-0.007602886	0.06285724	13.069305	0.003124968	0.21312238	10.77583	8.79×10^{-7}
NAc	-0.010115690	-0.16302591	10.585186	-0.005223113	-0.34851409	12.79812	3.08×10^{-5}

Tissue	TCF/LEF downstream		The others		pvalue
	Mean	Gene numbers	Mean	Gene numbers	
aINS	0.041	1046	0.021	25597	3.20×10^{-2}
vmPFC	0.020	1056	0.021	25593	8.83×10^{-1}
dlPFC	-0.021	1047	-0.032	25805	2.33×10^{-1}
OFC	-0.013	1060	-0.013	25916	9.56×10^{-1}
vSUB	-0.009	1060	-0.027	25927	8.70×10^{-2}
NAc	-0.018	1048	-0.007	25609	2.22×10^{-1}

Tissue	REST downstream		The others		pvalue
	Mean	Gene numbers	Mean	Gene numbers	
aINS	0.040	1339	0.044	25304	6.54×10^{-1}
vmPFC	0.020	1355	0.024	25294	5.91×10^{-1}
dlPFC	−0.022	1347	−0.005	25505	1.10×10^{-2}
OFC	−0.013	1353	−0.013	25623	9.84×10^{-1}
vSUB	−0.009	1358	−0.011	25629	8.76×10^{-1}
NAc	−0.017	1339	−0.036	25318	1.10×10^{-2}

Supplementary Table S4. Enriched GO terms in the gene sets of sertraline-treated cultured neurons.

Description	ID	NES	pvalue	p.adjust
Positive				
Postsynaptic neurotransmitter receptor activity	GO:0098960	2.411	4.72×10^{-9}	3.71×10^{-7}
Monoatomic ion gated channel activity	GO:0022839	2.081	1.00×10^{-10}	1.28×10^{-8}
Neuropeptide binding	GO:0042923	2.020	8.96×10^{-4}	1.08×10^{-2}
Glutamate receptor activity	GO:0008066	2.000	2.69×10^{-4}	4.21×10^{-3}
G protein-coupled amine receptor activity	GO:0008227	1.963	4.89×10^{-4}	6.68×10^{-3}
Cyclic nucleotide binding	GO:0030551	1.957	6.51×10^{-4}	8.50×10^{-3}
Structural constituent of synapse	GO:0098918	1.943	3.31×10^{-4}	4.91×10^{-3}
Serotonin receptor activity	GO:0099589	1.938	1.39×10^{-3}	1.54×10^{-2}
Neurotransmitter binding	GO:0042165	1.925	1.63×10^{-3}	1.74×10^{-2}
cAMP binding	GO:0030552	1.921	1.72×10^{-3}	1.83×10^{-2}
Fibroblast growth factor receptor binding	GO:0005104	1.917	1.81×10^{-3}	1.88×10^{-2}
Monoatomic ion channel activity	GO:0005216	1.905	1.00×10^{-10}	1.28×10^{-8}
Acetylcholine binding	GO:0042166	1.892	2.15×10^{-3}	2.14×10^{-2}
Glutamate receptor binding	GO:0035254	1.872	1.79×10^{-4}	3.04×10^{-3}
Alkali metal ion binding	GO:0031420	1.838	4.03×10^{-3}	3.46×10^{-2}
Glycine binding	GO:0016594	1.809	5.77×10^{-3}	4.40×10^{-2}
Syntaxin binding	GO:0019905	1.760	1.15×10^{-3}	1.31×10^{-2}
Salt transmembrane transporter activity	GO:1901702	1.588	3.12×10^{-7}	1.38×10^{-5}
Negative				
Immune receptor activity	GO:0140375	−2.016	1.35×10^{-6}	4.76×10^{-5}
Cytokine binding	GO:0019955	−1.928	1.60×10^{-6}	5.44×10^{-5}
Pattern recognition receptor activity	GO:0038187	−1.883	2.20×10^{-4}	3.59×10^{-3}
Structural constituent of chromatin	GO:0030527	−1.861	6.49×10^{-4}	8.49×10^{-3}
Integrin binding	GO:0005178	−1.851	5.71×10^{-6}	1.64×10^{-4}
Virus receptor activity	GO:0001618	−1.830	5.12×10^{-4}	6.94×10^{-3}
Sulfuric ester hydrolase activity	GO:0008484	−1.824	1.84×10^{-4}	3.12×10^{-3}
Fibronectin binding	GO:0001968	−1.808	4.00×10^{-4}	5.71×10^{-3}
Natural killer cell lectin-like receptor binding	GO:0046703	−1.796	2.18×10^{-3}	2.17×10^{-2}
Enzyme inhibitor activity	GO:0004857	−1.746	1.07×10^{-6}	3.87×10^{-5}
Cyclin-dependent protein serine/threonine kinase regulator activity	GO:0016538	−1.743	1.33×10^{-3}	1.48×10^{-2}
Apolipoprotein binding	GO:0034185	−1.743	4.56×10^{-3}	3.73×10^{-2}
CARD domain binding	GO:0050700	−1.726	2.77×10^{-3}	2.60×10^{-2}
Immunoglobulin binding	GO:0019865	−1.721	2.37×10^{-3}	2.31×10^{-2}
Structural constituent of eye lens	GO:0005212	−1.721	3.00×10^{-3}	2.77×10^{-2}
Single-stranded DNA helicase activity	GO:0017116	−1.715	3.59×10^{-3}	3.18×10^{-2}
Nucleoside binding	GO:0001882	−1.688	2.56×10^{-3}	2.46×10^{-2}
S100 protein binding	GO:0044548	−1.669	5.80×10^{-3}	4.42×10^{-2}
Cytokine receptor binding	GO:0005126	−1.650	1.65×10^{-4}	2.86×10^{-3}
Cardiolipin binding	GO:1901612	−1.628	6.71×10^{-3}	4.90×10^{-2}
Collagen binding	GO:0005518	−1.621	4.62×10^{-3}	3.75×10^{-2}
Protease binding	GO:0002020	−1.613	9.02×10^{-4}	1.08×10^{-2}
Glycosaminoglycan binding	GO:0005539	−1.572	6.94×10^{-4}	8.91×10^{-3}
Molecular function inhibitor activity	GO:0140678	−1.569	3.98×10^{-5}	8.91×10^{-4}
Carbohydrate binding	GO:0030246	−1.480	3.02×10^{-3}	2.78×10^{-2}

Supplementary References

1. B. Labonté, *et al.*, Sex-specific transcriptional signatures in human depression. *Nat Med* **23**, 1102–1111 (2017).

2. H. Abe, K. Abe, PCR-based profiling of transcription factor activity in vivo by a virus-based reporter battery. *iScience* **25**, 103927 (2022).
